# Cliques for the identification of gene signatures for colorectal cancer across population

**DOI:** 10.1186/1752-0509-6-S3-S17

**Published:** 2012-12-17

**Authors:** Meeta P Pradhan, Kshithija Nagulapalli, Mathew J Palakal

**Affiliations:** 1 School of Informatics, Indiana University Purdue University Indianapolis, IN, USA

## Abstract

**Background:**

Colorectal cancer (CRC) is one of the most commonly diagnosed cancers worldwide. Studies have correlated risk of CRC development with dietary habits and environmental conditions. Gene signatures for any disease can identify the key biological processes, which is especially useful in studying cancer development. Such processes can be used to evaluate potential drug targets. Though recognition of CRC gene-signatures across populations is crucial to better understanding potential novel treatment options for CRC, it remains a challenging task.

**Results:**

We developed a topological and biological feature-based network approach for identifying the gene signatures across populations. In this work, we propose a novel approach of using cliques to understand the variability within population. Cliques are more conserved and co-expressed, therefore allowing identification and comparison of cliques across a population which can help researchers study gene variations. Our study was based on four publicly available expression datasets belonging to four different populations across the world. We identified cliques of various sizes (0 to 7) across the four population networks. Cliques of size seven were further analyzed across populations for their commonality and uniqueness. Forty-nine common cliques of size seven were identified. These cliques were further analyzed based on their connectivity profiles. We found associations between the cliques and their connectivity profiles across networks. With these clique connectivity profiles (CCPs), we were able to identify the divergence among the populations, important biological processes (cell cycle, signal transduction, and cell differentiation), and related gene pathways. Therefore the genes identified in these cliques and their connectivity profiles can be defined as the gene-signatures across populations. In this work we demonstrate the power and effectiveness of cliques to study CRC across populations.

**Conclusions:**

We developed a new approach where cliques and their connectivity profiles helped elucidate the variation and similarity in CRC gene profiles across four populations with unique dietary habits.

## Background

Colon rectal cancer (CRC) is the third most commonly diagnosed cancer worldwide. It is the second leading cause of cancer death in the United States, and worldwide, nearly 608,000 deaths are reported every year due to CRC. The CRC incidence rate varies across the globe. For example, low incidence rates for CRC have been associated with Asian and African populations. Dietary and environmental factors have also been known to contribute to CRC patterns [1]. Therefore, we postulate that there are some common as well as some unique key gene signatures that can discriminate CRC across populations.

Due to the advent of high through-put technologies, a multitude of public domain expression datasets are now available for CRC research. These datasets are generated worldwide and deposited with the objective of identifying key molecules that play an important role in different stages of CRC. Gene-expression profiling and meta-analysis have been extensively used to: a) understand the mechanisms that drive a normal cell to become a cancer cell, b) understand different metastatic levels [2-6], and c) identify biomarkers [7]. Differentially expressed genes have been identified as biomarkers in leukemia, B-cell lymphoma, breast and lung cancers [8-11]. Gene signatures are a set of genes that might play an important role in a given disease. Using gene expression datasets, gene signatures were identified in different cancers [12-14]. First attempts to identify gene signatures from gene expression were done in breast cancer [10]. Genes combine together and act as pathways to perform a biological function and genes in a given pathway are co-expressed [15]. Large-scale efforts are being made to identify the biomarkers associated with specific pathways and biological function using gene expression profiles [16-21]. A single pathway can be deregulated by different mechanisms or combination of genes. Also, a set of genes can target one or many pathways. Gene signatures can help to identify these patterns in pathways and also the relationships among them [22]. First attempts for identifying gene signatures were done for breast cancer [10] and have since been used in various other cancers as well [12-14].

Network based approaches have been used to identify subnetwork markers (gene signatures) that are more reproducible than individual markers [23-25]. Functional modules extracted from networks are groups of genes with same functions [26]. The genes in the subnetworks are co-expressed (high/low) and they share more interactions among them, than with other genes in the larger network [27,28]. These functional modules can be used to identify both similar and unique biological characteristics among different species datasets [29] and are also considered to be subnetworks [30]. In protein-protein interaction networks, these functional modules are present as sub-graphs or tightly connected sub-graphs [31,32] and can be analyzed with respect to their individual characteristics using either Gene Ontology similarities or Pathway significance [33-35]. Identification of regulatory modules or gene subnetworks is important as they play critical roles in biological processes [36] and their associated pathways can provide potential targets for drug intervention in cancers [37].

Though gene signatures can improve understanding of a disease, identification of these signatures across populations is difficult, as gene expression is known to vary between populations [38]. Even though modules have been effectively used for the identification of gene signatures, this approach is computationally complex because the modules are open subnetworks [39], meaning that within a disease network, a very large number of modules will be identified [40]. Therefore, use of modules for comparing gene signatures across populations is computationally an intractable problem. Though attempts have recently been made to understand the difference in CRC between African-Americans and European-Americans [41] using a systems biology approach. However, not much work has been done in the area of gene signature identification across populations with respect to CRC.

Due to the complexity of gene signature identification, we propose the use of cliques as an alternative to modules for the comparison of gene signatures across populations. Cliques are closed, fully-connected subnetworks. The genes that are identified as part of these cliques are functionally related and highly co-expressed [33]. Since cliques are closed networks, they are both computationally tractable and more conserved in the biological networks [42]. A clique consists of molecules that can be associated with one or many pathways and these molecules are related with their Gene Ontologies [43]. A recent study reported the use of cliques in elucidating the mechanisms involved in breast cancer [44].

In this paper we have attempted to understand CRC gene signatures across four different populations: USA, Germany (GER), China (CHN), and Saudi Arabia (SA). The studies on each of these populations were conducted separately, and the data was downloaded from public repositories GEO http://www.ncbi.nlm.nih.gov/geo. For the study model, we hypothesized that tumors target biological modules that execute specific biological processes [45]. Since cliques are fully connected conserved subnetworks within biological networks, our hypothesis is that they are conserved across populations and can be understood as gene signatures. Therefore we propose to understand these cliques in CRC across populations. In this work we integrated the expression data along with network topological features and biological features. Cliques were then scored based on these features. Our work identified the common and unique cliques across populations that were important with respect to CRC. To identify the important cliques we analyzed the networks based on the following perspectives: (i) identification of genes from individual datasets based on *p-value*; (ii) construction of gene networks for each population; (iii) annotation of nodes and edges of networks with topological and biological features; (iv) identification of cliques across networks; (v) comparison of the cliques in all the networks based on their strength and connectivity profiles; and, (vi) evaluation of the cliques as gene signatures based on their biological significance in CRC.

## Results and discussion

**Figure 1:**
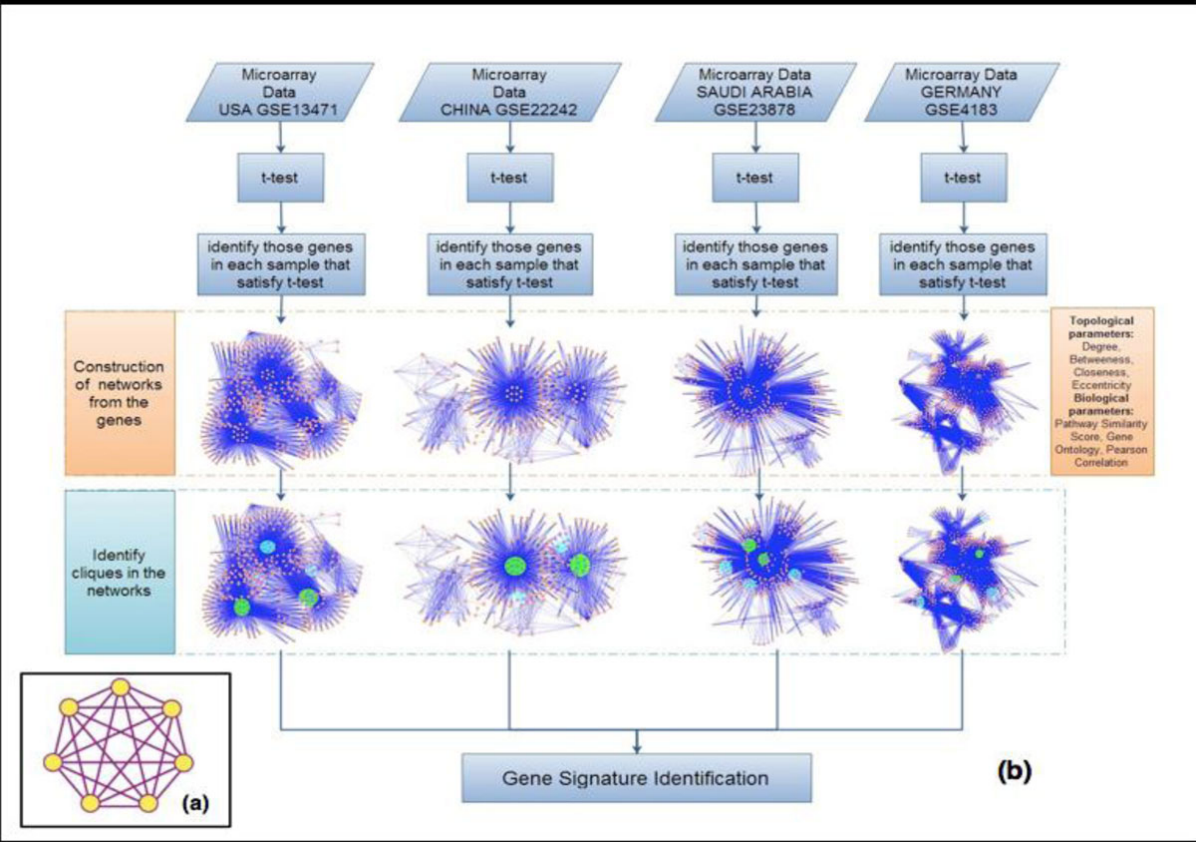
**Overall methodology to identify the unique and common cliques in the population network**. (i) Identify genes satisfying t-test in each data set. (ii) Construct networks for each dataset and annotate each node and edge in the network with its respective topological and biological features. (iii) Identify cliques of all sizes in each network and annotate each clique with its clique strength. Identify the maximum common size and highest scored clique as the seed across networks. (iv) Using the seed, identify the clique connectivity profile across networks. (v) Compare the clique connectivity profile (CCP) across network for commonality and uniqueness. (vi) Evaluate CCPs for their biological processes and pathways across networks and identify gene signatures for CRC across populations.

jIn order to decipher the gene signatures and identify the similarity-uniqueness among the four different populations of CRC (USA, GER, CHN, SA), we developed a methodology as described in Figure 1. Our methodology involved identifying genes in each dataset that satisfied the two sample *t*-test, construction of the gene networks using Human Protein Reference Database (HPRD) [46], obtaining the gene expression profiles (up- and down-regulated genes), identifying cliques in each dataset and comparing them across the populations, and connecting the cliques in each network to identify a Clique Connectivity Profile (CCP) and comparing them across populations.

### Data analysis

**Figure 2:**
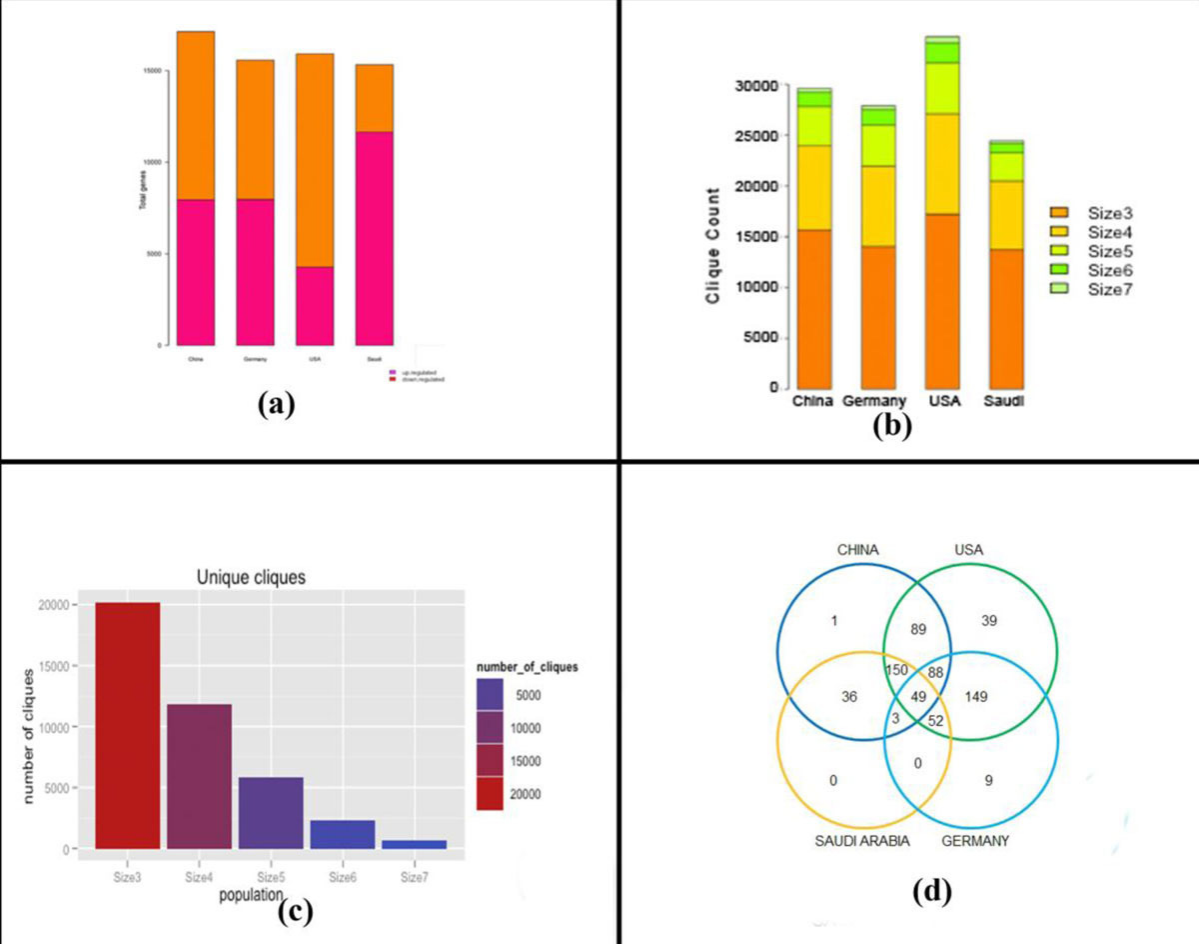
**(a) Gene expression profile for genes satisfying the t-test across populations**. SA showed the highest number of up-regulated genes, followed by GER, CHN, and then USA. (**b) **Clique distribution across population. USA had the highest number of cliques for all the sizes, while SA had the lowest number of cliques. CHN and GER had nearly same number of cliques of all sizes. (**c**) Total number of unique cliques of respective sizes identified across population. There was a large decrease in size 7 unique cliques identified in all the populations compared to numbers of cliques of other sizes. (**d**) The number of common cliques identified across all populations was 49. The cliques identified in SA overlapped with all the other populations.

The gene expression in all the four datasets was first normalized using the R-package RMA algorithm [47]. The two-sample *t*-test was used to identify the differentially expressed genes in each dataset. The genes satisfying the *t*-test (*p*-value < 0.05 and q-value with FDR < 0.1) in each dataset were then used to construct the networks. Figure 2(a) shows the profile of gene expression across the population dataset.

### Network construction

To construct the gene network for each population, we used only those genes that coded for proteins present in the HPRD database [46]. The networks were compared with respect to their node similarity. Table 1 shows the node (i.e., gene) similarity across the four populations. As shown in Table 1, a large number of genes were common among USA, CHN and GER, but there were fewer genes common with SA.

**Table 1:** Node similarity across population

Country (Total no. of nodes)	USA (7811)	CHN (7830)	SA (7182)
GER (7452)	6797	6815	6290
SA (7182)	6564	6587	--
CHN (7830)	7119	--	--

### Analysis of population specific networks

To analyze these population-specific networks with respect to their topological and biological features, these networks were first compared with the HPRD network for their interactions, degree, diameter, and average path length. Table 2 shows the results of this comparison. The average path-length is the overall ease with which the genes in the network communicate with each other. Though the degree and number of interactions differ for GER, USA, and SA with HPRD, the diameter and the average path lengths of these networks is in accordance with HPRD. Therefore these networks have the ability to generate functional complexes or modules and can be analyzed with respect to their biological processes.

**Table 2:** Comparison of population networks with HPRD network

Network	No. of interactions	Degree diameter	Av. Path length
HPRD	35706	7.79	4.22
CHI	27877	7.71	5.42
GER	25453	5.24	5.43
USA	28453	5.598	5.34
SA	24754	5.3	5.43

HPRD had the highest number of interactions. This illustrates that all four population networks are sub-networks within HPRD

For further analysis of the networks, Pearson Correlation Coefficients (PCC) were computed for each edge, and correlations greater than 0.6 and less than -0.6 were considered. This reduced the size and the complexity in the networks.

### Analysis of node strength based on topological properties

The node strength for each node in the population specific network was computed using the topological parameters - namely, eccentricity, closeness and betweenness (see method section, eqns. (i), (ii), (iii), and (iv)). The common high scoring nodes identified in all the population were: TP53, SRC, ESR1, SMAD3, GRB2, EP300, CREBBP, EGFR, SMAD2, and CSN2KA1. Of these, the transcription factors TP53, ESR1, SMAD3, and SMAD2 were identified as important in CRC [48]. GRB2 overexpression has also been identified in CRC [49] It was reported that binding of GRB2 with GAB2 plays an important role in CRC [50]. GAB2 additionally interacts with BCR-ABL, activating BCR-ABL-associated CRC significant pathways - specifically, PI3K-mTOR, RAS/RAF/MEK/ERK, and JAK-STAT [51]. EP300 has also been identified to be involved in CRC [52]. There were also a few unique top scoring nodes: ATXN1 for GER and PRKCA, PRKACA and UBQLN4 for SA. Not many references were available for UBQLN4 and ATXN1 in CRC, although ATXN1 has been associated with cancer pathways [53-55]. The topological analysis revealed top scoring genes that are common (known) as well as unique (not fully understood) with their significance in CRC across the four populations.

### Analysis of biological features for population specific networks

The biological feature analysis provided the edge strength for all the genes in all four population networks. The three biological features considered for the analysis were: PCC, Gene Ontology distance, and Pathway similarity score. The PCC had already been computed during the network construction process as described earlier (method section - equation (v)). The number of edges satisfying the PCC in each population network was: 15876 (USA), 12769 (CHN), 11664 (GER), and 9025 (SA). As it is known that, biological processes are essentially a series of events accomplished by one or more molecular functions. Each node in the network was associated with its biological processes. Gene Ontology distance was computed across an edge between two nodes in the network (method section: equation (*vi*)). The number of unique Gene Ontology biological processes terms identified in each of the population networks were: 2806 (CHN), 2801 (GER), 2674 (SA), and 2791 (USA). The following Gene Ontology biological processes were associated with maximum number of genes in all networks: GO: 0007165 (signal transduction), GO: 0006468 (protein phosphorylation), GO: 0006955 (immune response), and GO: 0055114 (oxidation-reduction process). Of these processes, signal transduction pathways are currently used as therapeutic targets in CRC [56], and immune response has been associated with CRC progression [57]. Since these biological processes are known to be important in CRC, we concluded that GO biological process should be a key feature for computing the EdgeStrength (method section - equation (vi)). Another key biological feature that we use was the Pathway similarity score between two nodes. The pathways were identified using the KEGG database [53,54], and the number of unique pathways identified for the respective populations were: 99 (CHN), 92 (USA), 54 (GER), and 87 (SA). There were a total of 105 unique pathways across all the four populations: forty-nine pathways were common to all four countries, thirty-five were common to three countries, seven were common to two countries, and five were unique to one country. The CRC pathways identified across all the countries were: Chemokine signaling pathways, Wnt-signaling pathway, MAPK signaling pathway, JAK-STAT pathway, Calcium-signaling pathway, ErbB signaling pathway, and Pathways in cancer [54,58,59]. The association of major CRC pathways with the nodes thus justified the use of the pathway similarity score as an essential feature for computing edge strength. Each node in the network was annotated with its pathway, and the pathway similarity score was computed across an edge of two nodes in the network (method section - equation (vii)).

Through these various analyses, we obtained the topological and biological features for all four populations to compute the NodeStrength and EdgeStrengths for their respective genes in the networks.

### Identification of cliques

Genes with similar expression patterns across various networks perform similar functions [27,28]. Both functional modules and interacting modules have similar co-expressed genes [60]. Based on this understanding, we designed an algorithm that identified the cliques (described in method section) in each population network. Figure 2(b) shows the distribution of the number of identified cliques of different sizes in each of the population-specific network. Figure 2 (c) shows the total number of unique cliques identified for all four populations. The largest number of cliques (all sizes included) was identified for USA and minimum for SA respectively. In this analysis, we considered only cliques of node size seven, as this size was found to be consistent across all four population networks, while cliques of higher sizes were not found across all the populations. For the specified clique size of seven, a total of 650 cliques were identified across the four populations. These cliques were then further analyzed with respect to their distribution in the different populations. There were 49 cliques common to all populations, while 20, 10, and 1 unique clique were identified in USA, GER, and CHN, respectively. Figure 2 (d) shows the Venn diagram for the distribution of size seven cliques across the four populations. The total number of genes identified in these cliques within each population network was: 126 (USA), 114 (CHN), 108 (GER), and 95 (SA). We identified 137 genes in total, with 57 of those genes common among all cliques across the populations.

### Analysis of cliques common across the populations

To understand the significance of the cliques across the populations, we first analyzed all the cliques with respect to their GO biological processes and pathway associations. The numbers of GO biological processes associated with cliques for each population network were: 247 (USA), 235 (GER), 222 (CHN), and 192 (SA), 247 (USA). GO terms with hyper-geometric *p-values < 0.05 *for each population were identified (method section- equation (xi)); GO: 0007165, GO: 0006468 and GO: 007049 were identified as the processes with the smallest hyper-geometric *p*-values across all cliques in all population networks. GO:0007165 was associated with signaling pathways, which have been identified as the targets for CRC [61]. GO: 007049 was associated with cell division cycle. Previous studies found genes involved in cell cycle, apoptosis, and invasion to play an important role in CRC [62]. GO: 0006468 was associated with protein phosphorylation. TGF-beta is a key pathway involved in CRC, and progression of this pathway is known to be dependent on protein phosphorylation [48,61,63]. The validation of these pathways with respect to involvement in CRC supports that clique nodes are involved in important GO biological processes, and these enriched GO terms are key features by which CRC can be evaluated across populations.

The identified cliques were further analyzed using GO Term Finder [64], and DAVID level 3 [65]. Cliques identified as significant (*p *< 0.05) were further evaluated based on literature. Table 3 shows the details of a few common cliques identified in all the population networks and their gene significance in CRC. It was also observed that some of the genes in these common cliques have been widely studied in terms of CRC, while others have relatively sparse available literature.

**Table 3:** Common cliques across the four population datasets

Clique	Enriched GOTerms	Processes(p-value)	Literature of CRC
EGFR, ESR1, GRB2, PTPN6, SHC1, SRC, PIK3R1	GO:0007173	EGFR signaling pathway (0.00253)	ESR1 [69]
	
	GO:0071363	Cellular response to growth	GRB2 [49]
	
	GO:0007105	Signal transduction (0.002)	EEGFR [49]

BRCA1, CREBBP, EP300, ESR1, SMAD2, SMAD3, TP53	GO:000637	Regulation of transcription from RNA polymerase II promoter (0.0044)	BRCA1 [73], CREBBP [72]
	
	GO:0033993	Response to lipid (0.00266)	SMAD2 [48]
	
	GO:0031325	Positive regulation of cellular metabolic process (0.00048)	P53 [48], EP300 [72]

CSN2, CSN3, CSN4, CSN5, CSN6, CSN7, CSN8, TP53	GO:000338	Protein deneddylation (1.7E-18)	
		
	GO:0044267	Cellular protein metabolic process (0.00029)	

DIS3, EXOSC2, EXOSC4, EXOSC5, EXOSC7, EXOSC8, EXOSC9, MPP6	GO:0045006	DNA deamination (9.47E-06)	EXOSC [81]
	
	GO:0006304	DNA modification (0.00192)	
	
	GO:0006402	mRNA catabolic process (0.00769)	

GO TERM FINDER AND DAVID level 3 (biological processes) were considered for analysis.

The clique {EGFR, ESR1, GRB2, PIK3R1, PTPN6, SHC1, SRC} in Table 3 was enriched in the following GO biological processes: EGFR signaling pathway, cellular response to growth, and signal transduction; most of the genes in this clique have been identified as significant in CRC. For example, targeted therapy using EGFR is currently available for CRC [66]. EGFR is a trans-membrane tyrosine kinase receptor belonging to HER family of cell surface receptor; it is triggered by ligands and leads to the activation of many intracellular signal transduction pathways (e.g., RAS, PI3K-AKT, STAT) [67] which are known to effect the activation of many transcription factors involved in cellular response (differentiation, apoptosis, proliferation, and migration). ESR1 is used as a epigenetic marker [64], while activation of GRB2/SOS, leads to a cytoplasmic phosphorylation cascade involving KRAS [68,69].

KRAS pathways are the targeted pathways in CRC [70], PTPN6 mutation has not been identified specifically in CRC, but it has been found in other cancers, including lymphoma and leukemia. Similarly, although SHC1 has not been identified directly in CRC, it has been identified in lung cancer [71].

The clique {BRCA1, CREBBP, EP300, ESR1, SMAD2, SMAD3, TP53} was enriched with the following GO biological processes: regulation of transcription, response to lipid, and positive regulation of cellular metabolic process. Again, these genes have all been studied in terms of CRC or other cancers. CREBBP and EP300 have been identified as prognostic markers for CRC [72], and EP300 is additionally been identified in lipid metabolism in CRC [65]. Some studies have identified BRCA1 [73], up-regulation of CREBBP [52,72], mutation of EP300, loss of SMAD2 signaling [74] in CRC. The TGF-Beta signaling pathway is known to play an important role as tumor suppressor and tumor promoters in CRC by activating the SMAD2/SMAD3 complex, which enters the nucleus to further regulate transcription [61,75]. Overexpression and mutation of TP53 has also been associated with CRC [76-78].

The clique {CSN2, CSN3, CSN4, CSN5, CSN6, CSN7A, CSN8, and TP53} is a part of signalosome of CSN9, which acts as protein kinase. The enriched GO biological processes were protein deneddylation and cellular protein metabolism. CSN-mediated protein deneddylation has been identified in the literature to promote Hedgehog-pathways, though it has not been reported specifically in CRC [79]. CSN3 has been identified as essential for cell proliferation in hepatocellular carcinoma, while CSN5 is known to be a regulator of TP53 and MDM2; additionally, CSN6 is known to be important for regulating DNA-damage-associated apoptosis and tumor genesis, as well as enhancing p53-mediated tumor suppression http://www.genedistiller.org. MDM2 has been identified as a probable therapeutic target for CRC [62,80].

In the clique {DIS3, EXOSC2, EXOSC4, EXOSC5, EXOSC7, EXOSC8, EXOSC9, and MPP6}, the enriched GO biological processes identified were: DNA modification, DNA deamination, and mRNA catabolic process. A form of DNA modification that is used to identify many cancers is DNA methylation in the promoter regions, which causes silencing of many genes [81]. DIS3, which has been identified in cancer genomes, stabilizes RNA and its translation into proteins [82], while EXOSC4 is involved in ribosome biogenesis and is highly up-regulated in CRC [83]. Though not much has been reported about the other genes in this clique with respect to CRC, they have been identified in other cancers.

One of the unique cliques identified for USA was {LSM1, LSM2, LSM3, LSM5, LSM6, LSM7, SMN1}. LSM1 is mapped on chromosome 8p11.2, which has been identified in both prostate cancer [78] and CRC. Although SMN1 has not been identified in cancer directly, it has been proposed to interact with BCL-2, which is associated with CRC, and has a high prognostic value [84,85]. From this analysis, it can be stated that common and unique cliques identified across population networks are involved in important biological processes in CRC. These cliques include genes that are both well-studied and less-studied in CRC, as well as those known to play a role in other cancers, indicating their importance in CRC networks and in better understanding CRC across populations. This analysis also demonstrates the importance of cliques in the CRC disease and can be used to understand the four population-specific networks.

### Analysis of pathways associated with genes in cliques for all populations

**Figure 3:**
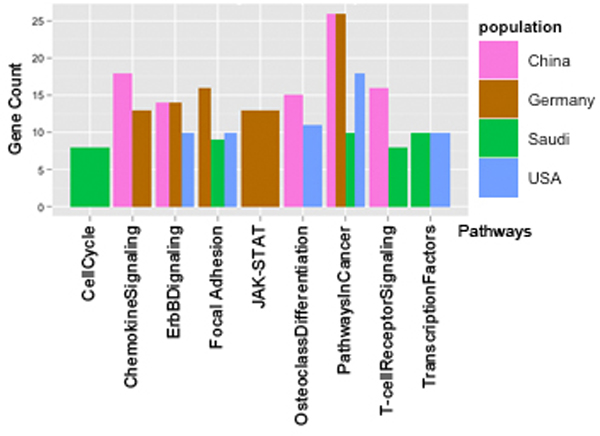
**Clique gene distribution in pathways across population**. More clique-genes were associated with Pathways in Cancer in population CHN, GER, USA than any other pathways for the same populations.

Cliques identified in the population-specific networks were further analyzed using the KEGG database for their pathway similarity score. Figure 3 shows the profile of pathways associated with the maximum number of genes in each network. This association varies across populations. For example, Pathways in Cancer is associated with the highest number of genes in all the populations - 26 (CHN), 26 (GER), 18 (USA), and 10 (SA);many of the different pathways that belong to the domain of Pathways in Cancer in the KEGG database were discussed in the previous section. Though JAK-STAT pathways were identified to be associated with clique-genes in all the population, the level of association, as defined by number of clique-genes identified, was higher in GER (9) than in SA (3). Similar observations of varying levels of association were made for many pathways, as can be seen in Figure 3.

Based on our analysis of the common cliques, unique cliques, and pathways associated with the cliques for all four populations, gene signatures for CRC can then be developed from the genes identified in these cliques. Specific gene signatures for each individual population could also be developed using the unique cliques that were identified in each population.

### Analysis of CliqueStrength

The parameter CliqueStrength was computed for all cliques in the population-specific networks based on both topological and biological features (method section - equation (ix)). Cliques associated with high CliqueStrength were considered important in these networks.

To assess the usefulness of the CliqueStrength parameter, we analyzed two top-scored cliques that were common or unique across the populations. Table 4 shows the top scored cliques in each network, their associated biological processes (GOTerm Finder, David level -3 terms), and the genes associated with each process. Table 4 additionally shows that the CliqueStrength of a common clique varies across population, as can be seen by comparing scores for the first clique in USA (5.91) and CHN (5.85); this is due to the fact that the parameter is a function of topological and biological features, which, along with network size, is variable across populations. The genes identified in the biological processes in the top scored cliques were either transcription factors (SMAD2, JUN, SMAD3), hub nodes, or those genes discussed in Table 5 that are known to play an important role in CRC. The top scoring clique identified in SA network was {MCM10, MCM2, MCM3, MCM4, MCM6, MCM7, ORC2L}. Interaction of highly expressed ORC2 and MCM6 is responsible for the initiation of DNA replication [46]. Moreover, ORC2L is not yet identified in CRC but is associated with breast cancer [43]. These results suggest that the top scored cliques indeed are associated with genes of significance in CRC.

**Table 4:** Top scored cliques in each population network

Cliques	Country	GOTerm, DAVID-level3	Genes Identified in GO Terms
EP300, ESR1, SP1, SMAD4, JUN, SMAD2, SMAD3	USA(5.91)	GO:0045595 Regulation of cell differentiation	EP300, SMAD2, JUN
	
	CHN(5.85)	GO:0045595 Regulation of cell differentiation	EP300, SMAD2, JUN
		
		GO:0048522 Positive regulation of cellular process	EP300, SMAD2, SMAD3, SMAD4, SP1, ESR1, JUN

CTNNB1, CREBBP, EP300, ESR1, SMAD2, SMAD3, SMAD4	GER (5.38) CHN (5.48) USA(5.341)	GO:0033993 Response to Lipid	EP300, SMAD2
		
		GO:0010769 Regulation of cell morphogenesis	EP300, SMAD2

PTPN11, CBL, SRC, PRKCA, SHC1, PTPN6, EGFR	SA(3.75)	GO:0007173 Epidermal growth factor Receptor signaling	EGFR, PRKCA, SHC, CBL, SRC, SHC1, PTPN11
		
		GO:0071363 Response to growth factor stimulus	PRKCA, CBL, SHC

MCM10, MCM2, MCM3, MCM4, ORC2L, MCM6, MCM7	SA(4.19)	GO:0006270 DNA-dependent DNA replication initiation	ORC2L, MCM6
		
		GO:0000082 G1/S transition of mitotic cell cycle	ORC2L, MCM6

CliqueStrength of each clique was represented in brackets with respect to the population in which it was identified

**Table 5:** Analysis of clique connectivity profile MaxCliques

Population	Result of GO TERMFINDER & DAVID level 3	Pathways (p-value)
USA	Positive regulation of cellular processes (1.2E-7)	Wnt Signaling (4.3E-7) Colorectal Cancer (2.3E-6)
	
	Regulation of cell proliferation (3.3E-5)	TGF-beta signaling (2.7E-6) Cell cycle (1.2E-5) Signal transduction (2.05E-5) Pathways in Cancer (1.7 E-3) Hunting disease (2.3E-2)

GER	Cell morphogenesis (3.1E-6)	ErbB signaling pathway (1.5 E-7)
	
	Cellular response to chemical stimulus (4.4E-06)	Focal adhesion (1.1E-6) GnRH signaling pathway (3.2E-3)
	
	Positive regulation of cellular process (1.1E-11)	JAK-STAT(1.1E-3) Neurotrophin signaling pathway (2.8E-5)

CHN	Positive regulation of cellular process (7.3E-11)	Wnt Signaling (5.1E-5)
	
	Regulation of cell differentiation(1.3E-4)	B cell receptor signaling pathway(2E-2)
	
	Regulation of growth(5.8E-04)	T cell receptor signaling pathway (3.9E-2)

The high scoring cliques were further considered as a seed to identify the clique connectivity profiles (CCP) for gene-signature identification. The overall connectivity using the top scoring clique can help to identify the CRC gene signature profile for a specific population.

### Discovering clique connectivity profile (CCP)

Cliques cannot carry out biological processes in isolation, but rather, they interact with other cliques in order to perform a biological process. These interactions can also help to identify the interacting pathways between cliques. Identifying a clique's connectivity profile (CCP) is important for better understanding the biological processes and pathways. To decipher how these cliques interacted in the network, we analyzed them based on their connectivity profile. In our algorithm, we considered the connectivity of cliques based on two parameters: (i) identification of common (links) genes, and (ii) CliqueStrength (based on topological and biological features- equation (*viii*)). The initial condition of the CCP algorithm was the identification of common genes across cliques. For this analysis, the connectivity between two cliques was computed based on the following two conditions: (a) maximum number of common genes across cliques and Highest CliqueStrength (MaxCliques), and (b) minimum number of common genes across cliques and Highest CliqueStrength (MinCliques), (nn < = 4, where nn = number of common genes across cliques).

### Analysis based on MaxCliques condition

**Figure 4:**
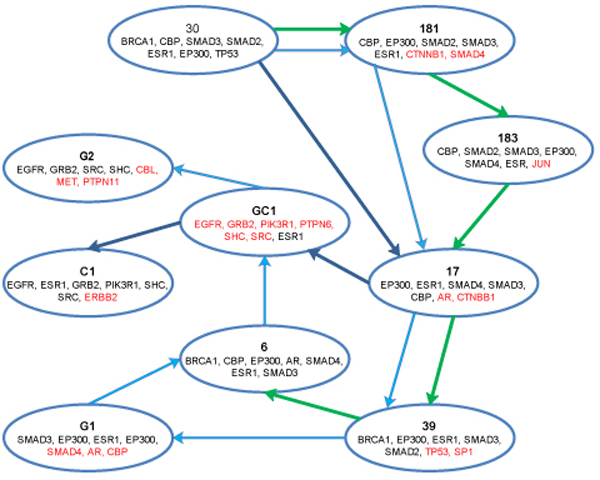
**Clique Connectivity Profile (MaxCliques)**. (Green-USA, Light blue-GER, Dark Blue-CHN). The figure depicts the clique connectivity profile for each of the populations. The seed was the same for all the populations, but the iteration considered maximum overlapping clique nodes and highest strength, resulting in overlapping nodes. The CCP diverges at three cliques, where the profile changes. The diverging cliques identified the genes that are both significant in CRC.

The clique with maximum CliqueStrength was selected as a seed, and the CCP was determined based on maximum common nodes and highest CliqueStrength until no new cliques could be added. For each iteration, the CliqueConnectivityScore was computed as described in the algorithm (Method section equation (x)). Figure 4 depicts the connectivity profile for one of the top common scoring cliques identified in USA, GER and CHN. These populations had cliques that were common and unique to all three connectivity profiles. Although the three CCPs shown in Figure 4 originated from the same seed, their connectivity profiles diverged at cliques #17 {EP300, ESR1, SMAD4, SMAD3, CBP, AR, CTNNB1}, #39 {BRCA1, EP300, ESR1, SMAD3, SMAD, TP53, SP1}, and #GC1 {EGFR, GRB2, PIK3R1, PTPN6, SHC, SRC, ESR1}. Clique#17 was the first divergent point where the profiles differed for CHN when compared to USA and GER. Expression of AR, which was included in Clique#17, has been associated with BRCA1 mutations in breast cancer [86]. Clique#39 includes BRCA1, whose mutations are associated with early-onset of colorectal cancer [48].

Transcription factors (SMAD2, SMAD3, P53, SMAD4, SP1, JUN) significant in CRC [48] were also identified both as hub nodes in our analysis and in these connectivity profiles. Using GOTerm finder and David-level 3, the biological processes for these CCPs were identified, and pathways associated with these CCPs were obtained from the KEGG database. Table 5 shows the enrichment with respect to GO biological processes and pathway analysis for these clique connectivity profiles.

The biological processes enriched in all three clique connectivity profiles (CCP) included Cellular process, and Cell differentiation. These were analyzed in earlier section of this paper and proved to be significant in CRC. The total numbers of pathways identified for genes present in the CCPs for each population were: 6 (USA), 22 (GER), and 25 (CHN). All pathways identified in USA and GER were also present in CHN. In USA, the pathway with lowest E-value was the Wnt signaling pathway; however, for GER, the ErbB signaling pathway was the lowest. Wnt signaling was identified in CHN along with the MAPK and Chemokine signaling pathways. These pathways are all known to be associated with biological processes in CRC [59,87-89].

**Figure 5:**
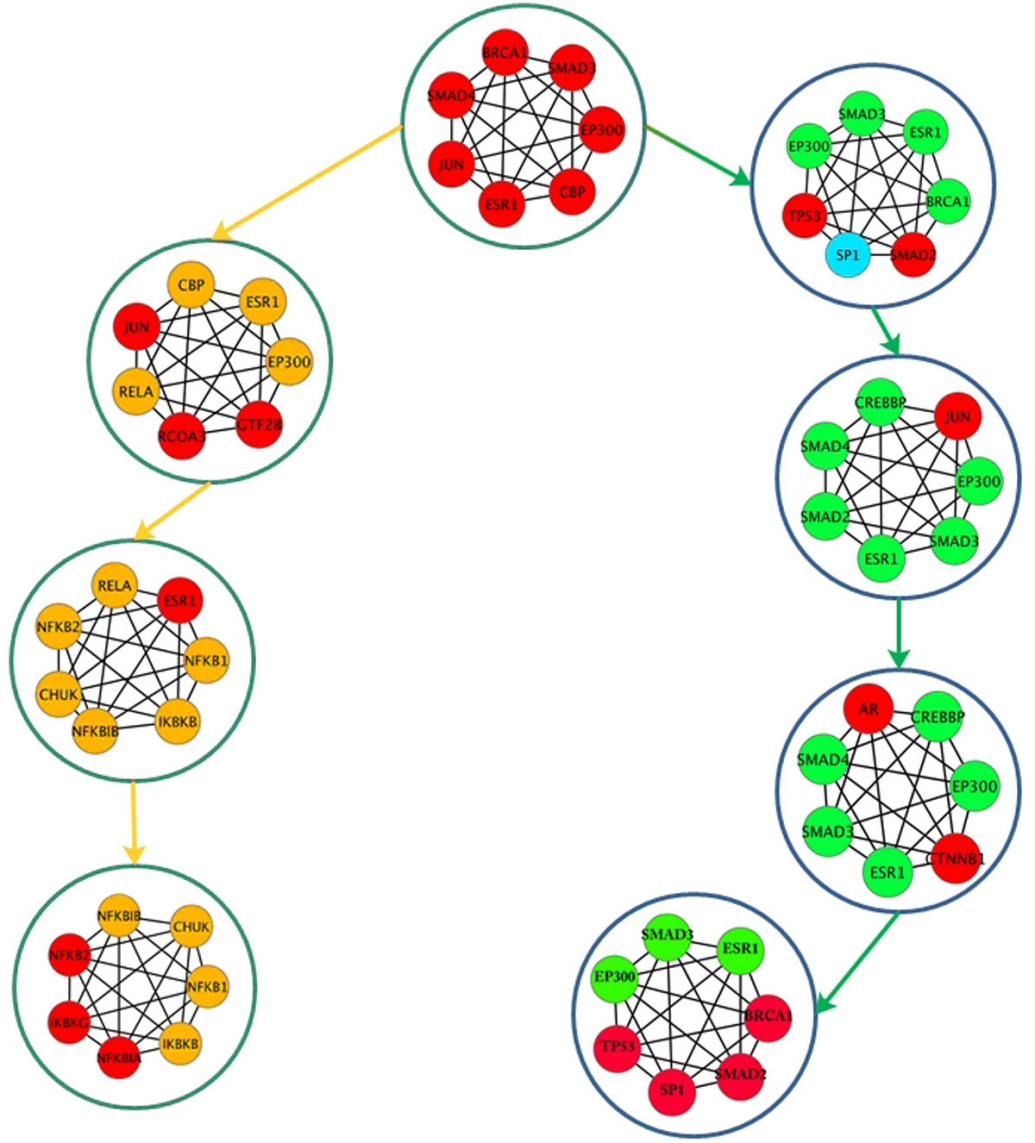
**Clique Connectivity Profile MaxCliques**. (Green USA, Yellow SA): This figure depicts the seed common to USA/SA. The iteration considered the identification of next clique by evaluating the maximum overlap and highest strength. It can be seen that the profile diverges at the seed itself. The genes identified in CCPs of both USA and SA are significant in CRC. This figure depicts the variability in the expression of genes across the population.

Figure 5 depicts the CCPs constructed using MaxCliques for top scored cliques common to USA and SA. From this figure it can be observed that the CCP diverges at the seed itself, indicating divergence in gene regulation between USA and SA. The biological processes associated with USA CCP were: positive regulation of cellular processes (1.2E-7), regulation of cell differentiation (1.1E-4), regulation of DNA binding (0.0062), and regulation of cell growth (1.3E-3) associated with CRC pathways. The biological processes associated with SA CCP were: regulation of metabolic process (4.8E-06), regulation of cell differentiation (6.9E-4), regulation of immune response (9.83E-09), and their associated pathways were: TGF-beta signaling, Wnt signaling, NOD-like receptor pathways, Toll-like signaling pathways, and MyD88 induced toll-like receptor pathways. Most of the biological processes identified across the CCP were common to both and are known to be associated with CRC, but the pathways associated with these processes were not overlapping. In the SA CCP, toll-like signaling pathway is identified. Toll-like receptor pathways play a key role in all the immune responses in CRC and are identified for cancer therapy [90]. While common cliques and pathways were identified for the populations of interest, subsequent analysis was also able to determine points of divergence within the connectivity profiles across all four populations.

This analysis depicts the importance of cliques and their connectivity profiles with respect to the important biological processes, and pathways and it helps to demonstrate the divergence of them across the four populations.

### Analysis based on MinCliques condition

**Figure 6:**
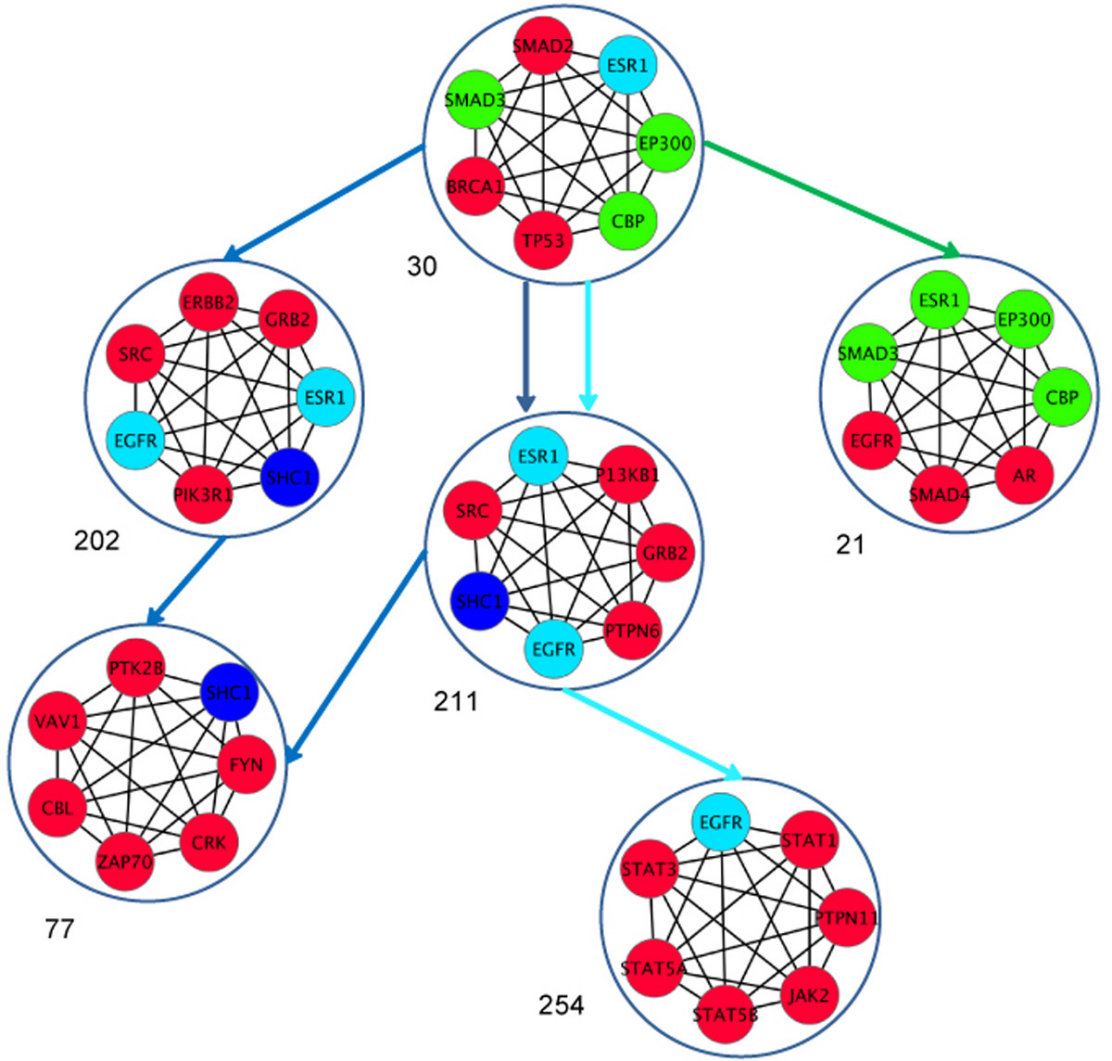
**Clique Connectivity Profile for MinCliques (Green-USA, Light blue-GER, Dark Blue-CHN)**. This figure depicts the CCP for USA, GER, and CHN that was identified using a common seed (same as in Figure IV). The algorithm considered minimum overlapping nodes with highest CliqueStrength. From this figure, we can see the CCP diverges at the seed itself for the three populations. In SA, we identified two cliques that have the same number of overlapping nodes with the seed and same clique strength. Therefore we see two gene signature profiles of SA, both of which end at the same clique.

Using the same seed as given in Figure 4, we found the CCPs for MinCliques as shown in Figure 6. The USA CCPs identifed a new clique that contains the EGFR gene, whereas for GER and CHN, the CCP identified the same divergent clique as shown in Figure 4 {EGFR, GRB2, PIK3R1, PTPN6, SHC, SRC, ESR1}. The CCP then diverged for GER and CHN. For CHN, the new connected clique contained the genes {ZAP70, VAV1, FYN, and CRK} while the GER clique had {STAT1, PTPN11, EGFR, JAK2, STAT5B, STAT5A, and STAT3}. ZAP 70 and VAV1 are known to be over-expressed in CRC [91], and STAT1, JAK2, and others are related to the JAK-STAT pathways associated with CRC. Figure 6 depicts the advantages and disadvantages of using a threshold for overlapping nodes to identify CCP - the altering the number of overlapping nodes.

When all clique sizes were considered for the MaxCliques algorithm, our study identified cliques with TAF1, TAF10, JUN, and FOS in all the populations. TAF1 is a regulator of apoptosis in cancer [92] and has been identified to be up-regulated in NCI-60 cell lines [93]. KRAS, which was present in a size three clique, was identified in USA, SA, and China populations. KRAS clique had its CCPs connected with the clique of BCL2 (size five-six). KRAS pathways are known to be associated with CRC [54]. The clique {MCM10, MCM2, MCM3, MCM4, MCM6, MCM7, ORC2L} identified in Table 4 was associated with cliques of CDKN1A (size four, five) in all the populations and down-regulation of CDKN1A plays a role in CRC [94]. The clique {DIS3, EXOSC2, EXOSC4, EXOSC5, EXOSC7, EXOSC8, EXOSC9, MPP6} identified in Table 3 does not have any CCP with any other size cliques in any population networks.

Genes present in cliques can form signatures for CRC specific to population. The CCP algorithm, if preceded with either MaxCliques or MinCliques, will still identify the important genes associated with CRC. Analysis of cliques of all sizes can identify the divergence in CCPs across population. As the definition of cliques is more stringent than that of modules, networks have fewer cliques than modules, allowing for more manageable analysis. Our analysis showed that CCPs can identify the commonality and divergence across populations. The ability of both cliques and CCPs to identify commonalities and divergences allows for them to be considered as gene signatures for CRC and can be evaluated further in the laboratory.

## Conclusions

In this paper we developed a methodology for identification of commonalities and variations in CRC across populations by evaluating cliques and their connectivity profiles. In this study, we considered four distinct populations across the world. We used both topological and biological features - specifically co-expression, GO distances for biological process, and pathway similarity scores - in our network analysis. We additionally introduced the concept of using cliques to capture gene signatures for CRC across populations. The methodology developed for joining cliques is powerful for finding the commonalities and divergences among populations with respect to their gene signatures. Using the CCP, we were able to capture important network components, including biological processes, pathways, and genes, and use these to elucidate the gene signature of CRC. The advantage of using cliques as opposed to functional modules is that although there are fewer cliques in a network, they are still able to capture the key gene signatures of a disease. Though the current study only applied the use of clique analysis to small datasets, we plan to validate the procedure in larger datasets. We additionally plan to make our CCP algorithm more stringent with respect to overlapping nodes. As our methodology is scalable with respect to annotation, different features such as static and dynamic profiles, literature score, and phenotypes can give in-depth stratification of CRC across populations. Comparison of all cliques (through their CCP) as gene signatures across populations may ultimately aid the advancement of personalized medicine and the identification of efficient drug targets.

## Methods

In order to decipher the gene signatures and identify the similarity/uniqueness among the four different populations of CRC, the following methodology, as illustrated in Figure 1, was adopted.

### Datasets

Four independent microarray studies available in the public domain repository GEO http://www.ncbi.nih.gov/geo were considered for this study. These studies were performed on the GPL 570 platform. The datasets from four different food habitats were considered - CHN, GER, SA and USA. These populations are quite distinct with respect to each other as there is less commonality in their diet and environmental conditions. The statistics for these different datasets are: (i) GER (GSE4183): 23 disease and 8 healthy control samples; (ii) SA (GSE23878): 35 disease and 24 healthy control samples; (iii) USA (GSE 13471): 4 disease and 4 healthy control samples; and, (iv) CHN (GSE22242): 1 disease and 1 healthy control sample. Raw data in each case was processed using the RMA algorithm in R Bio conductor http://www.r-project.org[47]. The normalized datasets were then analyzed by two-sample *t*-test. The genes satisfying the *t*-test (*p-value *< 0.05 and q-value with FDR < 0.1) were further considered for differential expression analysis across the populations.

### Construction of the interaction network

For the above genes the population specific networks, were constructed using the protein-protein interactions obtained from the HPRD database [46].

### Analysis of population specific networks

Networks were first analyzed individually based on their topological and biological features. Each node in the network was first annotated for its topological properties, with the edges providing the biological significance.

### Node strength based on topological properties

Using the statistical computing tool R, each node in the network was scored for its Degree, Eccentricity, Closeness, and Betweenness properties. Degree was defined by the number of connections a given node had with other nodes in the network. Eccentricity of a node was defined by the ease with which it could be accessed by all the other nodes in the network. Eccentricity of a node *v *was calculated by computing the shortest path between the node *v *and all other nodes in the network as,

(i) $$Eccentricity\left(E_{ecc}\left(v\right)\right)=\frac{1}{\text{max}\left\{dist\left(v,w\right):w\in V\right\}}$$

Where w represents the number of nodes in set V of nodes and has the shortest distance to node v.

Closeness of a node *v *is the average of the shortest path between the node *v *and all other nodes in the network and was given by,

(ii) $$Closeness\;\left(C_{clso}\left(v\right)\right)=\frac{1}{\sum_{w\in V}dist\left(v,w\right)}$$

Betweenness of a node *v *is the inverse of the ratio of total number of shortest paths from node *s *to node *t *given by *σ_st _*to the number of total paths passing through node *v *(*σ_st _*(*v*)). This was computed as,

(iii) $$Betweenness\;\left(B_{bet}\left(v\right)\right)=\sum_{s\neq v\neq t}\frac{\sigma_{st}\left(v\right)}{\sigma_{st}}$$

Each of the above features was computed and normalized to obtain the node strength for *v*, which was given as,

(iv) $$NodeStrength_{v}=\frac{\left(Degree+E_{cc}+C_{clso}+B_{bet}\right)}{4}$$

### Edge strength based on biological properties

The edge strength was defined as the weight assigned to an edge connecting the two nodes (*v_i_, v_j_*) in the network. Edge strength was computed based on three biological features: PCC, Gene ontology distance, and pathway similarity score. PCC was used as a similarity measure between the two nodes as it identified the co-expressed genes, which encode interacting proteins and help in understanding cellular patterns [95], in the network.

The PCC was calculated for nodes (*v_i_, v_j_*) as,

(v) $$\mathrm{PearsonCor}r_{\mathrm{vi}\mathrm{vj}}=\frac{\sum_{k=1}^{n}\left(vi_{k}-vi_{mean}\right)\left(vj_{k}-vj_{mean}\right)}{\sqrt{\sum_{k=1}^{n}{\left(vi_{k}-vi_{mean}\right)}^{2}{\left(vj_{k}-vj_{mean}\right)}^{2}}}$$

Where *vi_mean_, vj_mean _*of the sample is means for the genes i and j, and n is number of samples.

The genes in the network were annotated based on GO biological process and evaluated for their similarity. The GO distance similarity for nodes (*v_i_, v_j_*) was computed as [96]:

(vi) $$\mathrm{GeneOntologyDistance}\left(v_{i}v_{j}\right)=\frac{\#\left(GO\left(G_{i}\right)\text{Δ}GO\left(G_{j}\right)\right)}{\#\left(GO\left(Gi\right)\cup GO\left(G_{j}\right)\right)+\#\left(GO\left(Gi\right)\cap GO\left(G_{j}\right)\right)}$$

Where, Δ is the symmetric set difference, and *GO*(*G_i_*) is the number of *GO *annotations for *v_i_*. Similarly, we computed *GO*(*G_i_*)) for *v_j_*. If the GO distance between (*v_i_, v_j_*) was less than 1.0, they were considered interacting. The interacting nodes are considered for constructing the network.

The Pathway similarity score was computed using pathways in KEGG database [53,54]. Each gene was annotated with its associated pathway, and the gene-pathway similarity score was computed as follows:

Let (*v_i_, v_j_*) represent the two nodes in the network. Let *P_N _*represent a set of pathways where gene *v_i _*is present, and *P_M _*represent the set of pathways where gene *v_j _*is present. *P_common _*then equals the number of common pathways identified in *P_N _*and *P_M_*, and *Unique *equals the unique number of pathways present in *P_N _*and *P_M_*. The pathway similarity score between (*v_i_, v_j_*) is defined as:

(vii) $$\mathrm{PathwaySimilarityScor}e_{\left(v_{i}v_{j}\right)}=\frac{\frac{Pcommon}{P_{N}}+\frac{Pcommon}{P_{M}}}{Unique\left(P_{N}+P_{M}\right)}$$

The three biological features were further normalized, and each interaction in the network was scored based on the average score for each of the features and given as,

(viii) $$\mathrm{EdgeStrengt}h_{v_{i}v_{j}}=\frac{PearsonCorr_{Norm}+GeneOntologyDistance_{Norm}+PathwaySimilarityScore_{Norm}}{3}$$

### Identification of cliques

Cliques are fully connected, conserved, and co-expressed in the networks [33,42]. We developed a graph-based approach to identify cliques in the networks with the purpose of understanding them as gene signatures across population. A clique was defined as a fully connected graph, as shown in Figure 1 (a). Let *G *= (*V, E*) be any arbitrary undirected graph with *V *= {1,2,3 ... *N*} as its vertices, and *E *= {(1, 2), (1, 3), ..., (1, *N*)}, the set of corresponding edges. A clique *C *is a sub-graph of *V *such that *C *∈ *V *and every vertex of *C *in the sub-graph is connected to all the other *C *- 1 vertcies. Each population network was then analyzed for cliques of various sizes, ranging from 3 to *M *nodes. For our analysis, *M *= 7. The strength of a clique was defined based on the associated node strength (eqn. *(iv)*) and edge strengths *(viii)*), and computed as:

(ix) $$\mathrm{CliqueStrengt}h_{i}=\sum_{Node\;\;j=1}^{n}\sum_{Edge\;\;k=1}^{e}NodeStrength+EdgeStrength$$

We used the greedy algorithm to first identify three-node cliques in the networks as a seed. The seed was then used for identifying cliques of higher sizes, ranging from four to seven nodes.

### Clique connectivity profile algorithm (CCP)

To understand the profile of the cliques across population, we developed an algorithm to discover the connectivity profile of the cliques based on the number of common nodes. Our hypothesis for this connectivity rule was that cliques with common nodes may have similar pathways and Gene Ontology biological processes. Each clique may traverse the network by taking different paths. Identification of the clique connection profile (CCP) was important to understanding the gene signature of CRC as the interacting genes in these cliques might be important for a function in a given biological process. The CCP algorithm annotated each clique with its total CliqueStrength (equation *(ix*)), and then identified its closest clique connection based on the number of common nodes and CliqueStrength. This CCP algorithm iteratively progressed until no new clique could be added to the path. The clique connectivity strength was computed as,

(x) $$\mathrm{CliqueConnectivityScor}e_{i,j}=\sum \frac{CliqueStrength_{ij}}{2}$$

The CCP algorithm first identified the clique (for similar size) with highest strength common to all the population. Using this as a seed, the algorithm proceeded ultimately produced a network of cliques that provided the gene signatures that are present across the populations for CRC. The smaller size cliques were added to this network of cliques. These CCPs could then be used to understand the commonality and uniqueness as gene-signatures in CRC across populations.

### Statistical evaluation of cliques using gene enrichment analysis

Hyper-geometric distribution based on *p-value *was used for identifying the significance of GOTerms in the network and cliques [97] and was computed as,

(xi) $$p-\mathrm{value}=\sum_{i=x}^{N}\frac{\left(\begin{array}{c} g\\ gg \end{array}\right)\left(\begin{array}{c} G-g\\ n-gg \end{array}\right)}{\left(\begin{array}{c} G\\ n \end{array}\right)}$$

where, significance of a given GOTerm *x, gg *genes in the n genes of cliques, and that is associated to *g *genes from *G *genes in the population network. GO Terms with *p*-values less than 0.05 were further used for analyzing the biological significance of the cliques.

## List of abbreviations

CRC: Colorectal cancer; CCP: clique connectivity profile; GO: Gene Ontology; GER: Germany; SA: Saudi Arabia; CHN: China; PCC: Pearson correlation coefficient.

## Competing interests

The authors declare that they have no competing interests.

## Authors' contributions

MPP: conceptualizing and developing methodology, writing and analysis of all the algorithms, writing manuscript.

KN: Data collection and data filtering, drawing figures, critical reading and editing of the manuscript.

MJP: PI of the project, conceptualizing the objective, writing manuscript, valuable inputs at all times.
